# Ongoing monkeypox virus outbreak, Portugal, 29 April to 23 May 2022

**DOI:** 10.2807/1560-7917.ES.2022.27.22.2200424

**Published:** 2022-06-02

**Authors:** Mariana Perez Duque, Sofia Ribeiro, João Vieira Martins, Pedro Casaca, Pedro Pinto Leite, Margarida Tavares, Kamal Mansinho, Luís Miguel Duque, Cândida Fernandes, Rita Cordeiro, Maria José Borrego, Ana Pelerito, Isabel Lopes de Carvalho, Sofia Núncio, Vera Manageiro, Corrado Minetti, Jorge Machado, Joana M Haussig, Roberto Croci, Gianfranco Spiteri, Ana Sofia Casal, Diana Mendes, Tiago Souto, Sara Pocinho, Teresa Fernandes, Ana Firme, Paula Vasconcelos, Graça Freitas

**Affiliations:** 1 Directorate of Information and Analysis, Directorate-General of Health, Lisbon, Portugal; 2 ECDC Fellowship Programme, Field Epidemiology path (EPIET), European Centre for Disease Prevention and Control (ECDC), Solna, Sweden; 3 National Plan Sexually Transmitted Infections and HIV, Directorate-General of Health, Lisbon, Portugal; 4 Department of infections, Department of Infectious Diseases, Centro Hospitalar Universitário de São João, Porto, Portugal; 5 EPI Unit, Instituto de Saúde Pública da Universidade do Porto, Porto, Portugal; 6 University of Porto Medical School, Porto, Portugal; 7 Infectious Diseases and Tropical Medicine Service, Centro Hospitalar Lisboa Ocidental, EPE/Hospital de Egas Moniz, Lisbon, Portugal; 8 Hygiene and Tropical Medicine Institute/NOVA University of Lisbon, Lisbon, Portugal; 9 Hospital Garcia de Orta, Almada, Portugal; 10 CheckpointLX, Grupo de Ativistas em Tratamentos (GAT), Lisbon, Portugal; 11 Sexual Transmitted Diseases Clinic, Dermatovenereology Department, Centro Hospitalar e Universitário de Lisboa Central, Lisbon, Portugal; 12 Department of Infectious Diseases, National Institute of Health Dr. Ricardo Jorge, Lisbon, Portugal; 13 ECDC Fellowship Programme, Public Health Microbiology path (EUPHEM), European Centre for Disease Prevention and Control (ECDC), Solna, Sweden; 14 European Centre for Disease Prevention and Control (ECDC), Solna, Sweden; 15 Directorate of Health Promotion and Disease Prevention, Directorate-General of Health, Lisbon, Portugal; 16 Division for Communication and Public Relations, Directorate-General of Health, Lisbon, Portugal; 17 Support Unit of National Health Authority and the Emergency Management in Public Health, Public Health Emergencies Operations Centre, Directorate-General of Health, Lisbon, Portugal; 18 National Health Authority, Directorate-General of Health, Lisbon, Portugal

**Keywords:** monkeypox, outbreak, surveillance

## Abstract

Up to 27 May 2022, Portugal has detected 96 confirmed cases of monkeypox. We describe 27 confirmed cases (median age: 33 years (range: 22–51); all males), with an earliest symptom onset date of 29 April. Almost all cases (n = 25) live in the Lisbon and Tagus Valley health region. Most cases were neither part of identified transmission chains, nor linked to travel or had contact with symptomatic persons or with animals, suggesting the possible previously undetected spread of monkeypox.

Human monkeypox (MPX) is a zoonotic disease caused by the monkeypox virus (MPXV) [1,2], which is endemic in several African countries [3,4]. Since the first ever case of human monkeypox was confirmed in Portugal on 17 May 2022, Portugal has become one of the most affected countries of the evolving outbreak outside endemic areas [5]. A multidisciplinary team was established to confirm the outbreak, investigate its origin and chains of transmission, and implement control measures. In this study, we describe the preliminary results of the outbreak investigation and the epidemiological characteristics of 27 confirmed cases.

## Outbreak detection

On 3 May 2022, five males with atypical skin ulcerative lesions with similar body distribution presented at Centro Hospitalar Universitário de Lisboa Central sexually transmitted infections (STI) outpatient clinic and at the GAT-CheckpointLX, a community based STI clinic for men who have sex with men (MSM). The lesions appeared predominantly in the genital area (perianal, scrotum and lining of the penis) as whitish-coloured lesions, which evolved with the formation of a central crust. All lesions were at the same clinical stage. Two cases had papules with similar characteristics on the trunk and limbs, but not more than 20 papules in total. The lesions in the intertriginous areas were sometimes ulcerated, which were the most painful according to the cases.

A broad list of differential diagnoses was considered, and many infections were ruled out including herpes simplex virus (HSV-1/2) infection, human immunodeficiency virus (HIV) infection, early syphilis, chancroid, gonorrhoea and chlamydia. On 16 May, following an alert on the European Centre for Disease Prevention and Control (ECDC) EpiPulse platform from the United Kingdom (UK) about a positive case of monkeypox in an individual with similar genital lesions, laboratory tests for orthopoxviruses were conducted and an emergency management team was established. On 17 May, the National Institute of Public Health (Instituto Nacional de Saúde Doutor Ricardo Jorge, INSA, IP) provided real-time PCR laboratory confirmation of the first three cases of MPX. The Portuguese General Directorate of Food and Veterinary Medicine laboratory of animal health was contacted upon the detection of these first cases.

## Timeline of the outbreak and descriptive epidemiology

Up to 27 May 2022, 96 cases of MPX have been confirmed in Portugal, accordingly to our case definition [6] (Table 1). Of these, 41 cases had data on date of symptom onset and exposure. An epidemic curve (Figure) shows the first cases in Portugal had symptom onset as early as 29 April; cases continued to be diagnosed throughout the analysis period (29 April–23 May 2022). The epidemic curve also shows different exposure routes, including attendance at specific venues, i.e. saunas used for sexual encounters, travel abroad (Spain, UK and Brazil) during the incubation period [7] and contact with non-Portuguese nationals. Only one case was a contact of another confirmed case.

**Table 1:** Working case definition for monkeypox [6], Portugal, 29 April–23 May 2022

Case classification	Definition
Suspected case	A person of any age with a rash (macular, papular, vesicular or pustular; generalised or localised) AND/OR anogenital complaints (including ulcers), with sudden symptom onset since 15 March 2022, unexplained by other differential diagnoses AND one or more of the following signs/symptoms: fever of sudden onset (≥ 38.0°C), asthenia, myalgia, backache, headache, lymphadenopathy.
Probable case	A person of any age who meets the suspected case criteria AND one or more of the following conditions: • contact with a suspected, probable or confirmed case of MPXV infection within 21 days before onset of symptoms, • a person who had multiple or anonymous sexual partners within 21 days before symptom onset, • hospital admission because of a clinical condition consistent with a suspected case, • travel history to MPX-endemic countries within 21 days before symptom onset.
Confirmed case	A person with a laboratory-confirmed MPX infection (real-time PCR-positive test result and/or nucleotide sequencing) in a clinical sample.

MPX: monkeypox; MPXV: monkeypox virus.

**Figure:**
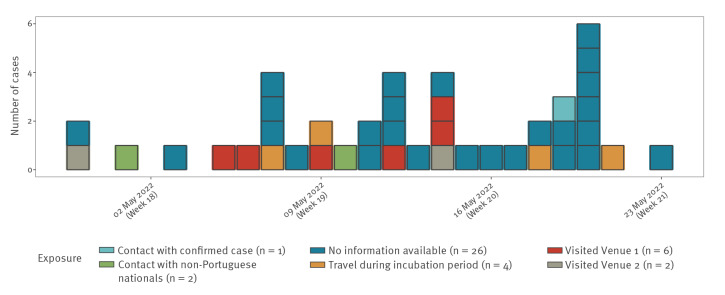
Confirmed monkeypox cases by date of symptom onset and exposure, Portugal, 29 April–23 May 2022 (n = 41^a^)

^a^ Only cases with data available for both date of symptom onset and exposure.

‘Visited Venue 1/2’ refers to confirmed cases who have been linked because of visiting the same venues. ‘No information available’ refers to no travel or contact data available. Travel to Spain, United Kingdom and Brazil was reported during the incubation period.

We obtained information on demographic characteristics, clinical presentation, and exposure from all 96 cases through face-to-face and phone interviews, using standardised case-investigation forms. We collected in-depth information on a subset of 27 confirmed cases and summarised the sociodemographic and clinical characteristics of the cases for which information from laboratory and epidemiological investigation was available (Table 2). The following analyses were based on these 27 confirmed cases.

**Table 2:** Demographic and clinical characteristics of confirmed monkeypox cases, Portugal, 1–23 May 2022 (n = 27)

Variables	Confirmed monkeypox cases
	n = 27
Sex	
Male	27
Female	0
Age (years)	
20–29	7
30–39	13
40–49	3
50–59	1
Unknown	3
Residence	
Lisbon and Tagus Valley	25
North	1
Algarve	1
Symptoms^a^	
Exanthema	14
Inguinal lymphadenopathy	14
Fever	13
Asthenia	7
Headache	7
Genital ulcers	6
Genital vesicles	6
Anal ulcers	5
Myalgia	5
Anal Vesicles	4
Cervical lymphadenopathy	4
Axillary lymphadenopathy	2
HIV infection^a^	
Yes	14
Exposures during the 21 days before onset of symptoms^a^	
Travel abroad	4
Contact with animals	3
Contact with people with similar symptoms	1
Hospital admission	
Yes	3

^a^ Some cases did not report on certain symptoms or clinical features. Missing information for fever, asthenia, headache, cervical lymphadenopathy, axillary lymphadenopathy (n = 1 case). Missing information for HIV infection (n = 1 case). Missing information for myalgia (n = 2 cases); for exanthema and genital ulcers (n = 12 cases); for inguinal lymphadenopathy (n=13 cases); for anal ulcers and anal vesicles (n = 14 cases); for contact with animals (n = 4 cases); for contact with people with similar symptoms (n = 17 cases).

Cases resided mainly in the Lisbon and Tagus Valley (LVT) health region (n = 25), with one also occurring in North Region and one in Algarve. Ages ranged from 22 to 51 years (median: 33 years) with the majority aged 30–39 years (n = 13). All cases were male. Considering exposure in the 21 days preceding symptom onset, very few cases (1/10) reported contact with people presenting similar symptoms or history of travel abroad (4/27). Where data were available, almost all cases identified themselves as men who have sex with men (MSM) (18/19) whereas one case reported having sex with only women.

During the 21-day period before the onset of symptoms, most cases (14/16 with available data) reported having had sex with multiple partners. Of the 27 cases, six cases reported attendance at a sauna in the LVT health region, one case reported frequenting a sauna in the UK and four cases reported travelling abroad. Three had contact with animals (two cases with cats and one case with pigs).

The most common symptoms were exanthema (n = 14), inguinal lymphadenopathy (n = 14), fever (n = 13), genital ulcers (n = 6). A total of 14 cases had an HIV infection. Three cases required hospitalisation because of their clinical condition, of which two have since been discharged. No deaths have been registered up to 27 May 2022. One middle-aged case reported prior vaccination against smallpox.

## Laboratory investigations

Since the clinical picture of MPX has been atypical in this outbreak (Table 2), with lesions reported to start in the genital area instead of the face, additional laboratory tests were performed to exclude other infectious diseases at the time of detection. A total of 190 samples obtained from 145 suspected cases (133 males and 12 females, which includes the 96 confirmed cases) were collected in 23 governmental and non-governmental healthcare facilities throughout Portugal between 5 and 27 May 2022. The specimens tested included swabs of lesion surfaces, exudate and/or crusts from lesions in the palms, genital area, and/or oral mucosa. Diagnosis with PCR in real time was based on the detection of the *Orthopoxvirus* genus gene *rpo18*, followed by Sanger sequencing of PCR products. Specific identification of MPXV was performed as previously described [8-10]. On 20 May, the virus clade was identified by metagenomics performed directly from a confirmed case sample using an Oxford Nanopore Technologies (ONT) MinION sequencer [11].

Of 145 suspected cases, 96 (66.2%) tested positive for MPXV DNA by PCR, with virus identity being confirmed by sequencing in the first three cases. Preliminary analysis of the virus genome sequence showed that it belongs to the West African clade [11,12], and is closely related to viruses previously imported from Nigeria to the UK, Israel, and Singapore in 2018 and 2019 [13,14].

## Early control measures and public health response

The Public Health Emergencies Centre and the Health Authorities in Portugal established an emergency management team to coordinate the response, investigate the outbreak, and perform contact tracing. One of the goals was to identify the event that introduced the virus into Portugal. Contact tracing has been difficult, as a large proportion of cases engaged in sex with multiple and/or anonymous sex partners. For confirmed cases, home isolation was recommended until lesions fade away, including exclusion of work (sick leave). The self-monitoring of contacts was recommended for 21 days from the date of last exposure (direct contact) to a case, per the World Health Organization (WHO) close contact definition [15]. A strategy for post-exposure prophylaxis of contacts with a poxvirus-derived vaccine is being considered. Healthcare workers have been advised to adhere to standard contact precautions, hand hygiene and barrier nursing through use of personal protective equipment (PPE) including gloves, face mask, gown, and goggles.

Other measures in place have included revisiting the first cases reported and – in close collaboration with clinicians – clarification of possible transmission chains and places of exposure. Active case finding using established standard case definitions together with prompt sample collection (lesion specimens for active cases and serum for retrospective cases) for laboratory diagnosis has been well-accepted among clinicians through practical procedures disseminated through the healthcare delivery units (public and private) and throughout the health authorities in all regions. Retrospective case finding is currently under discussion, for which serological studies will have a key role to document a previous MPX infection. Upon a decision, the case definition will be updated.

In addition, public health authorities engaged with the LGBTQI+ community – particularly community leaders – on risk communication and social mobilisation of the community after the first few cases to ensure that information on the infection signs and symptoms and how to reduce transmission was promptly shared.

## Discussion

Human MPX remains endemic in some African countries, though outbreaks have occurred outside this continent [3,4]. A past outbreak in 2003 in the United States was linked to importation of infected rodents [16,17]. In 2018 [16] and 2021 [18], travel-associated outbreaks were reported in the UK. This is the first MPX outbreak detected in Portugal.

The first detected cases appear to be mostly among MSM aged 30–39, living with HIV and having a mild form of disease. Thus far, no severe cases have been reported. The epidemic curve shows that most cases were not part of identified chains of transmission, nor were linked to travel or had contact with symptomatic persons or with animals. In our sample, the earliest symptom onset date was 29 April. Our findings, consistent with results from investigations in the UK [19], raise the hypothesis of possible undetected spread of MXPV occurring in Europe at least since early April and potential importation into Portugal. Early risk communication and targeted prevention approaches have been aimed at LGBTQI+-identifying people living in Portugal, with careful consideration to ensure a non-stigmatising approach. In addition, it is of utmost importance to raise awareness among healthcare professionals to detect suspected cases.

This outbreak, with ongoing cases in several other countries worldwide besides Portugal, highlights the importance of strengthening epidemic intelligence mechanisms to promote early detection of atypical and uncommon clinical conditions that require public health interventions, especially in the context of zoonotic diseases. MPX outbreaks in endemic areas usually do not extend beyond a few transmission cycles, and person-to person transmission is rarely reported [20]. The MPX outbreak in Portugal shows sustained transmission among a susceptible demographic group that has not been exposed to smallpox vaccination, which was mandatory in Portugal until 1977. This could suggest the waning or absence of cross-protective immunity provided by the smallpox vaccine.

There are two genetic groups (clades) of MPXV, West and Central African, the former usually associated with milder illness [2]. Preliminary genetic data from our study suggest importation from West Africa. Although MPXV is classified as moderately transmissible, studies have described an increasing genetic adaptation to human host, with enhanced potential for human-to-human transmission [21,22]. Further genetic characterisation of MPXV isolates from Portugal and other European countries is needed to elucidate the origin and disease dynamics of this outbreak.

We hypothesised that MPX has been circulating below the detection of the surveillance systems. Although some cases have a clear epidemiological link, the lack of an identified exposure in others raises unanswered questions. Prior studies have suggested a potential role of HIV coinfection [18]. Our study has some limitations, such as completeness of data and sample size, but individual determinants of transmissibility and infection susceptibility are still under investigation.

## Conclusion

Sustained human-to-human transmission in Portugal and other countries highlights MXPV as an emerging orthopoxviral infection, particularly after smallpox eradication. This study serves as a first step in understanding human MPX spread in a naive population in a non-endemic country and provides information to tailor preventive measures and risk communication. Our findings underscore the importance of atypical clinical presentations of human MPX and highlights the need for further and continued epidemiological investigations and research.

## References

[1] Durski KN McCollum AM Nakazawa Y Petersen BW Reynolds MG Briand S Emergence of Monkeypox - West and Central Africa, 1970-2017. MMWR Morb Mortal Wkly Rep. 2018;67(10):306-10. 10.15585/mmwr.mm6710a5 29543790 PMC5857192

[2] McCollum AM Damon IK . Human monkeypox. Clin Infect Dis. 2014;58(2):260-7. 10.1093/cid/cit703 24158414 PMC5895105

[3] Ladnyj ID Ziegler P Kima E . A human infection caused by monkeypox virus in Basankusu Territory, Democratic Republic of the Congo. Bull World Health Organ. 1972;46(5):593-7. 4340218 PMC2480792

[4] Di Giulio DB Eckburg PB . Human monkeypox: an emerging zoonosis. Lancet Infect Dis. 2004;4(1):15-25. 10.1016/S1473-3099(03)00856-9 14720564 PMC9628772

[5] European Centre for Disease Prevention and Control (ECDC). Epidemiological update: Monkeypox multi-country outbreak. Stockholm: ECDC; 25 May 2022. Available from: https://www.ecdc.europa.eu/en/news-events/epidemiological-update-monkeypox-multi-country-outbreak

[6] Directorate-General for Health. Orientação Técnica 004/2022. Abordagem de casos de infeção humana por vírus Monkeypox (VMPX). [Approach to cases of human infection by Monkeypox virus]. Lisbon: DGS; 31 May 2022. Portuguese. Available from: https://www.dgs.pt/normas-orientacoes-e-informacoes/orientacoes-e-circulares-informativas/orientacao-n-0042022-de-31052022-pdf.aspx

[7] European Centre for Disease Prevention and Control (ECDC). Risk assessment: Monkeypox multi-country outbreak – 23 May 2022. ECDC: Stockholm; 2022. Available from: https://www.ecdc.europa.eu/en/publications-data/risk-assessment-monkeypox-multi-country-outbreak

[8] Kurth A Achenbach J Miller L Mackay IM Pauli G Nitsche A . Orthopoxvirus detection in environmental specimens during suspected bioterror attacks: inhibitory influences of common household products. Appl Environ Microbiol. 2008;74(1):32-7. 10.1128/AEM.01501-07 17965204 PMC2223225

[9] Shchelkunov SN Shcherbakov DN Maksyutov RA Gavrilova EV . Species-specific identification of variola, monkeypox, cowpox, and vaccinia viruses by multiplex real-time PCR assay. J Virol Methods. 2011;175(2):163-9. 10.1016/j.jviromet.2011.05.002 21635922 PMC9628778

[10] Nitsche A Ellerbrok H Pauli G . Detection of orthopoxvirus DNA by real-time PCR and identification of variola virus DNA by melting analysis. J Clin Microbiol. 2004;42(3):1207-13. 10.1128/JCM.42.3.1207-1213.2004 15004077 PMC356842

[11] Isidro J, Borges V, Pinto M, Ferreira R, Sobral D, Nunes A, et al. First draft genome sequence of Monkeypox virus associated with the suspected multi-country outbreak, May 2022 (confirmed case in Portugal). [Accessed: 28 May 2022]. Available from: https://virological.org/t/first-draft-genome-sequence-of-monkeypox-virus-associated-with-the-suspected-multi-country-outbreak-may-2022-confirmed-case-in-portugal/799

[12] Isidro J, Borges V, Pinto M, Ferreira R, Sobral D, Nunes A, et al. Multi-country outbreak of Monkeypox virus: genetic divergence and first signs of microevolution. [Accessed: 28 May 2022]. Available from: https://virological.org/t/multi-country-outbreak-of-monkeypox-virus-genetic-divergence-and-first-signs-of-microevolution/806

[13] Mauldin MR McCollum AM Nakazawa YJ Mandra A Whitehouse ER Davidson W Exportation of monkeypox virus from the African continent. J Infect Dis. 2022;225(8):1367-76. 10.1093/infdis/jiaa559 32880628 PMC9016419

[14] Yinka-Ogunleye A Aruna O Dalhat M Ogoina D McCollum A Disu Y CDC Monkeypox Outbreak Team . Outbreak of human monkeypox in Nigeria in 2017-18: a clinical and epidemiological report. Lancet Infect Dis. 2019;19(8):872-9. 10.1016/S1473-3099(19)30294-4 31285143 PMC9628943

[15] World Health Organization (WHO). Surveillance, case investigation and contact tracing for Monkeypox. Interim guidance, 22 May 2022. Geneva: WHO. [Accessed: 26 May 2022]. Available from https://www.who.int/publications/i/item/WHO-MPX-surveillance-2022.1

[16] Petersen E Kantele A Koopmans M Asogun D Yinka-Ogunleye A Ihekweazu C Human Monkeypox: epidemiologic and clinical characteristics, diagnosis, and prevention. Infect Dis Clin North Am. 2019;33(4):1027-43. 10.1016/j.idc.2019.03.001 30981594 PMC9533922

[17] Centers for Disease Control and Prevention (CDC) . Update: multistate outbreak of monkeypox--Illinois, Indiana, Kansas, Missouri, Ohio, and Wisconsin, 2003. MMWR Morb Mortal Wkly Rep. 2003;52(24):561-4. 12816106

[18] Ogoina D Iroezindu M James HI Oladokun R Yinka-Ogunleye A Wakama P Clinical course and outcome of human monkeypox in Nigeria. Clin Infect Dis. 2020;71(8):e210-4. 10.1093/cid/ciaa143 32052029

[19] Adler H Gould S Hine P Snell LB Wong W Houlihan CF NHS England High Consequence Infectious Diseases (Airborne) Network . Clinical features and management of human monkeypox: a retrospective observational study in the UK. Lancet Infect Dis. 2022;S1473-3099(22)00228-6. 10.1016/S1473-3099(22)00228-6 35623380 PMC9300470

[20] Reed KD Melski JW Graham MB Regnery RL Sotir MJ Wegner MV The detection of monkeypox in humans in the Western Hemisphere. N Engl J Med. 2004;350(4):342-50. 10.1056/NEJMoa032299 14736926

[21] Reynolds MG Carroll DS Karem KL . Factors affecting the likelihood of monkeypox’s emergence and spread in the post-smallpox era. Curr Opin Virol. 2012;2(3):335-43. 10.1016/j.coviro.2012.02.004 22709519 PMC9533834

[22] Bunge EM Hoet B Chen L Lienert F Weidenthaler H Baer LR The changing epidemiology of human monkeypox-A potential threat? A systematic review. PLoS Negl Trop Dis. 2022;16(2):e0010141. 10.1371/journal.pntd.0010141 35148313 PMC8870502

[23] Hobson G Adamson J Adler H Firth R Gould S Houlihan C Family cluster of three cases of monkeypox imported from Nigeria to the United Kingdom, May 2021. Euro Surveill. 2021;26(32):2100745. 10.2807/1560-7917.ES.2021.26.32.2100745 34387184 PMC8365177

